# Supplementary material for: (E)-Piplartine Isolated from Piper pseudoarboreum, a Lead Compound against Leishmaniasis
Source: Foods. 2020 Sep 7;9(9):1250. doi: 10.3390/foods9091250 (PMC7554920; doi:10.3390/foods9091250)
Supplement: Supplementary file 1 [file foods-09-01250-s001.pdf]

Electronic Supporting Information

(*E*)-Piplartine isolated from *Piper pseudoarboreum*: a Lead Compound against Leishmaniasis

Juan C. Ticona [1,2], Pablo Bilbao-Ramos [3], Ninoska Flores [2], M. Auxiliadora Dea-Ayuela [3,4], Francisco Bolás-Fernández [3], Ignacio A. Jiménez [1,*] and Isabel L. Bazzocchi [1,*]

[1] Instituto Universitario de Bio-Orgánica Antonio González and Departamento de Química Orgánica, Universidad de La Laguna, Avenida Francisco Sánchez 2, 38206 La Laguna, Tenerife, Spain
[2] Instituto de Investigaciones Fármaco Bioquímicas, Facultad de Ciencias Farmacéuticas y Bioquímicas, Universidad Mayor de San Andrés, Avda. Saavedra 2224, Miraflores, La Paz, Bolivia
[3] Departamento de Parasitología, Facultad de Farmacia, Universidad Complutense de Madrid, Plaza Ramón y Cajal s/n, 28040-Madrid, Spain
[4] Departamento de Farmacia, Bioquímica y Biología Molecular, Universidad CEU-Cardenal Herrera, Avda. Seminario s/n, 46113-Moncada, Valencia

Contents

S2 ^{1}H and ^{13}C NMR spectra for compound **1**
S3 ^{1}H and ^{13}C NMR spectra for compound **2**
S4 ^{1}H and ^{13}C NMR spectra for compound **3**
S5 ^{1}H and ^{13}C NMR spectra for compound **4**
S6 ^{1}H and ^{13}C NMR spectra for compound **5**
S7 ^{1}H and ^{13}C NMR spectra for compound **6**

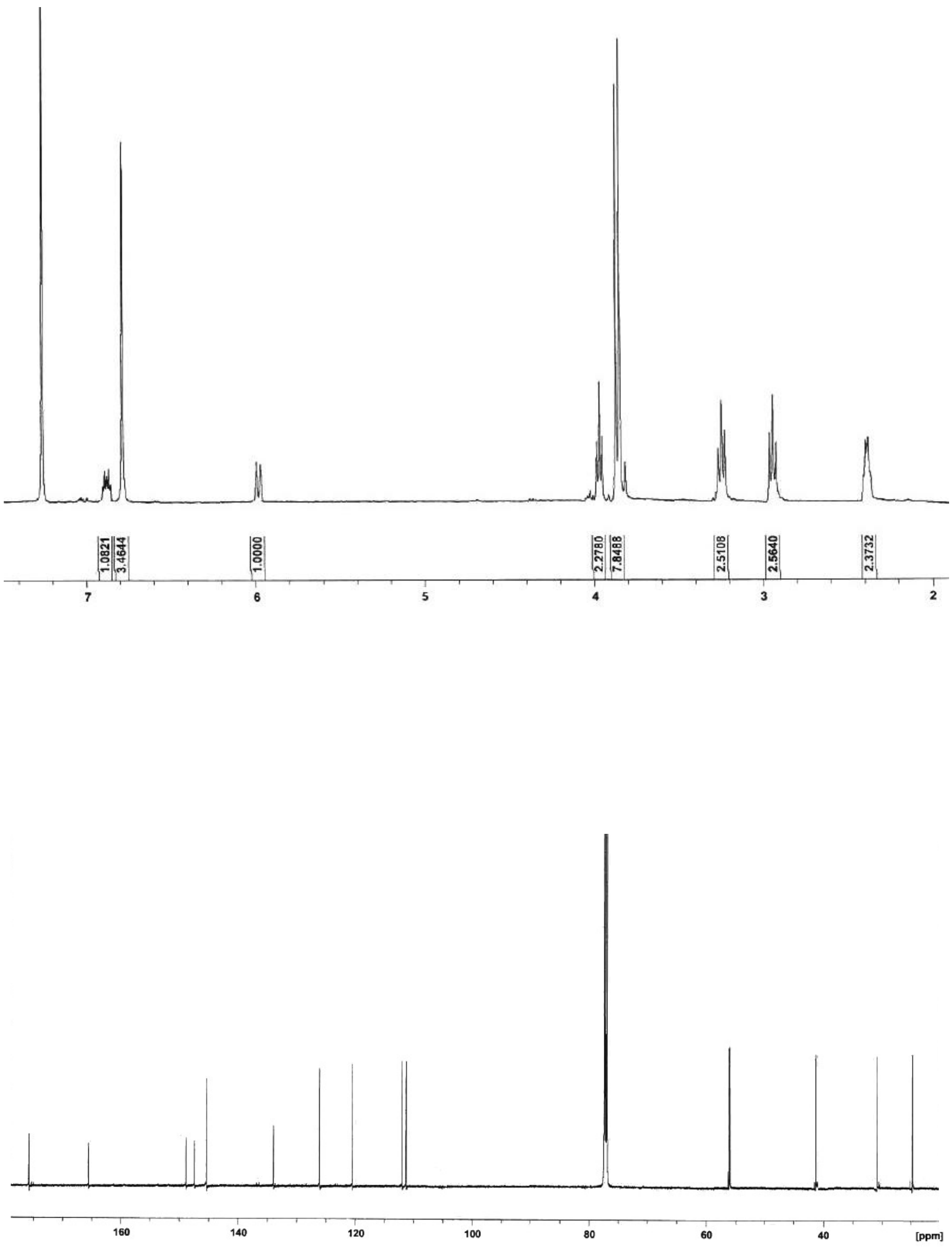

S2. ^{1}H and ^{13}C NMR spectra (400 MHz and 100 MHz, respectively, $CDCl_3$) of compound **1**.

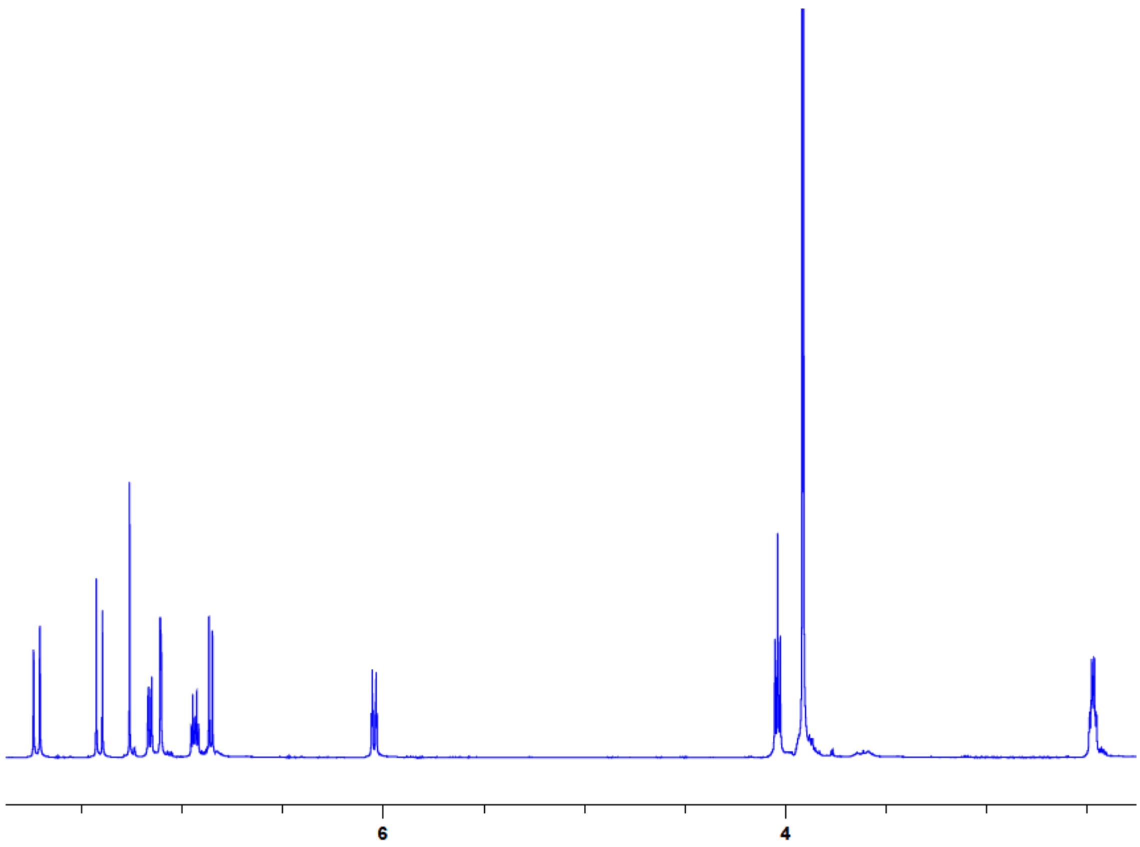

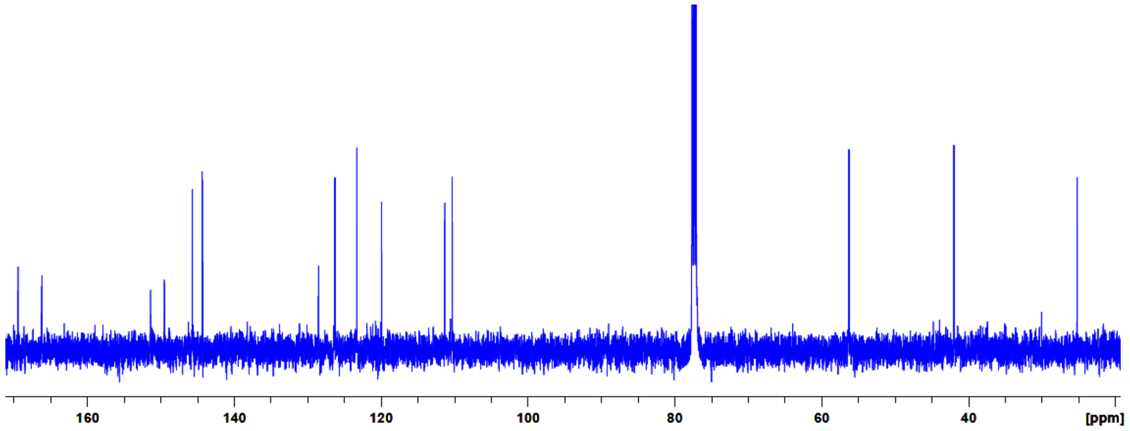

S3. ^{1}H and ^{13}C NMR spectra (500 MHz and 125 MHz, respectively, $CDCl_3$) of compound **2**.

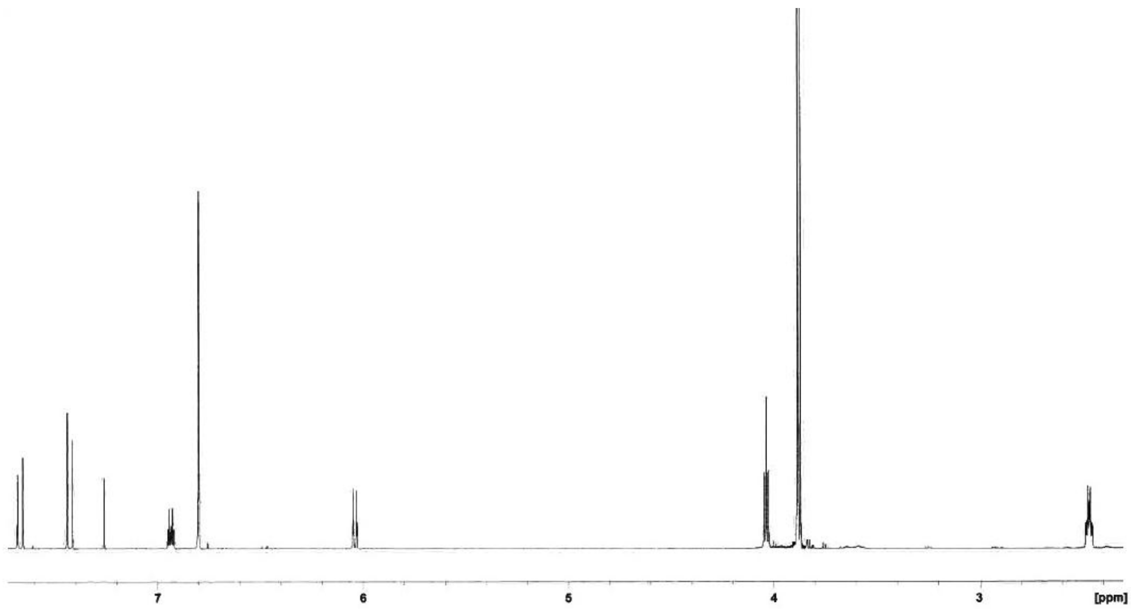

^{1}H NMR spectrum (400 MHz, solvent $CDCl_3$) of derivative **3**.

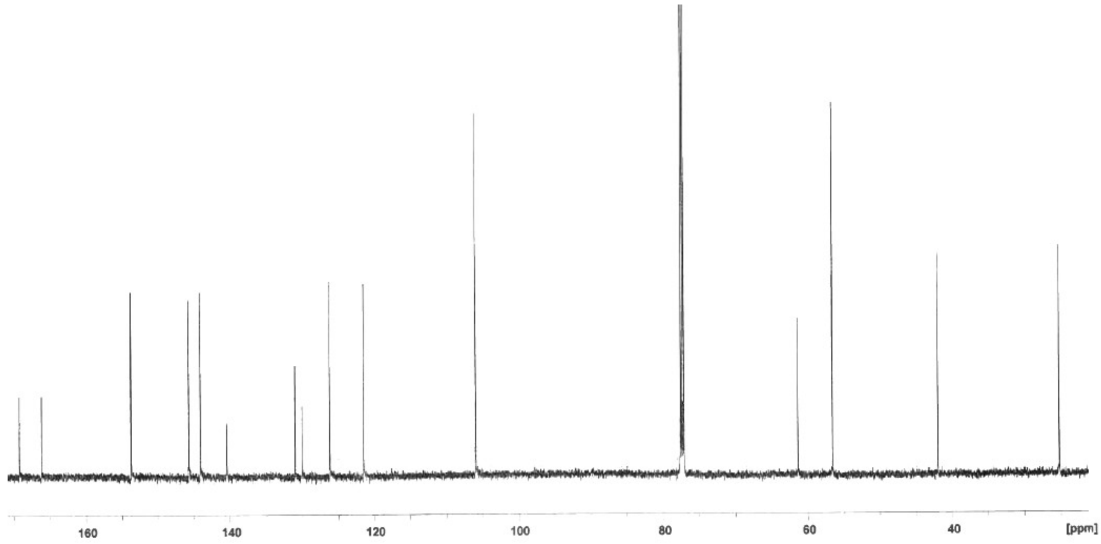

S4. ^{1}H and ^{13}C NMR spectra (400 MHz and 100 MHz, respectively, $CDCl_3$) of compound **3**.

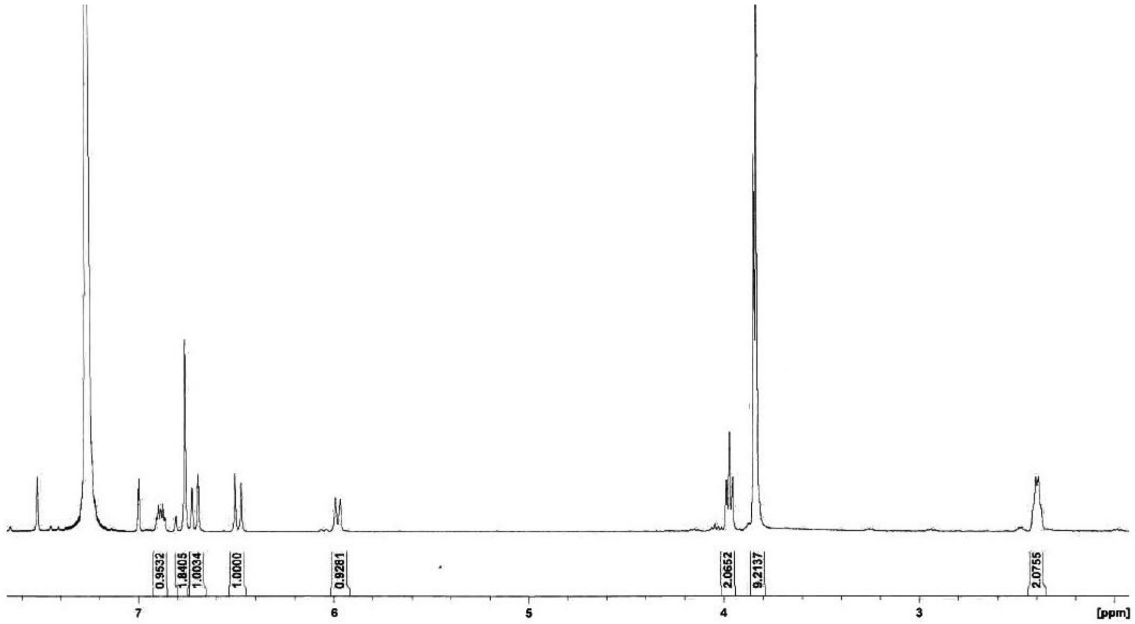

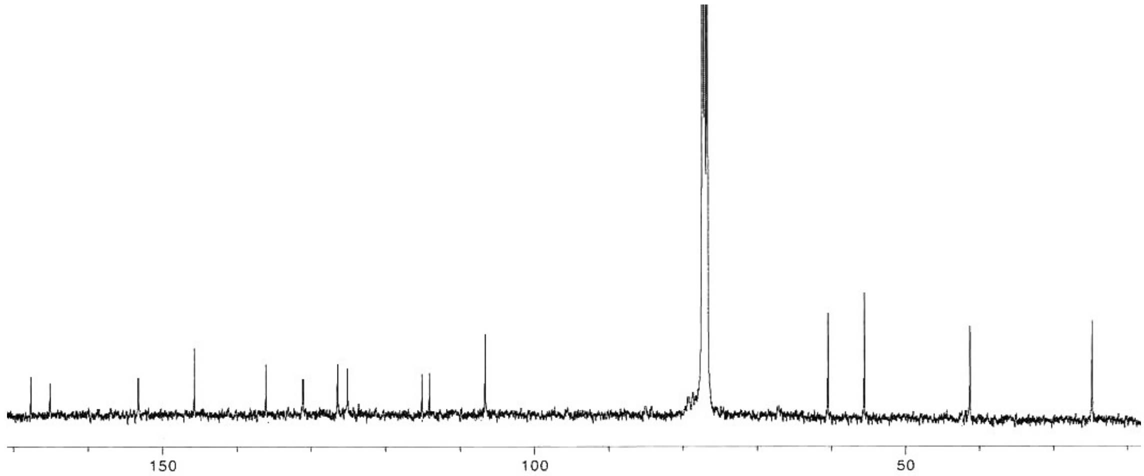

S5. ^{1}H and ^{13}C NMR spectra (400 MHz and 100 MHz, respectively, $CDCl_3$) of compound **4**.

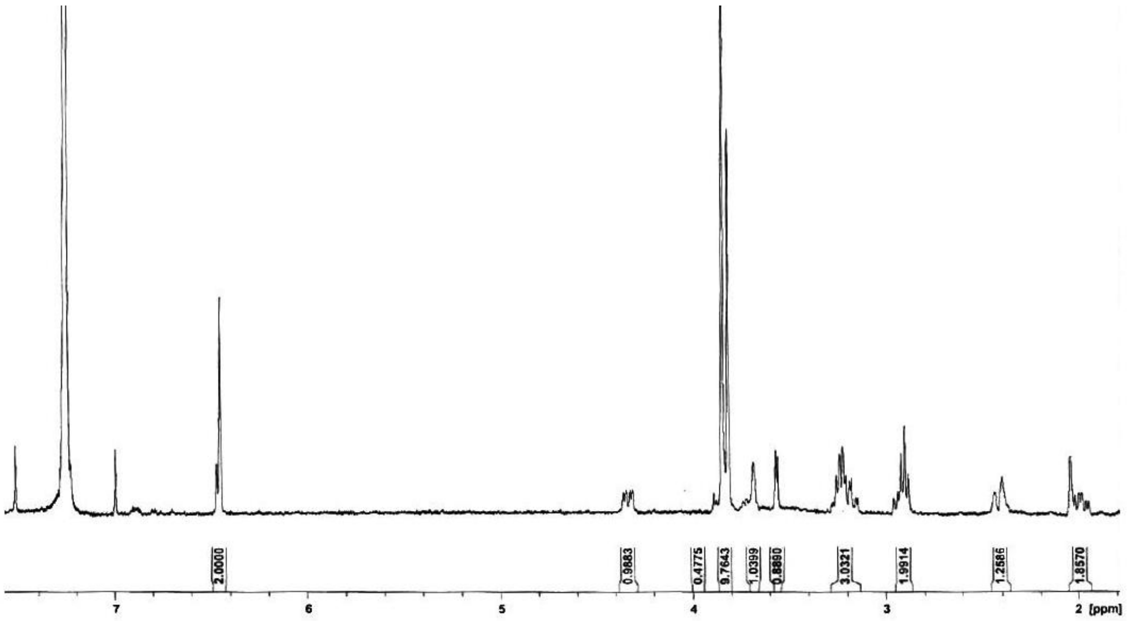

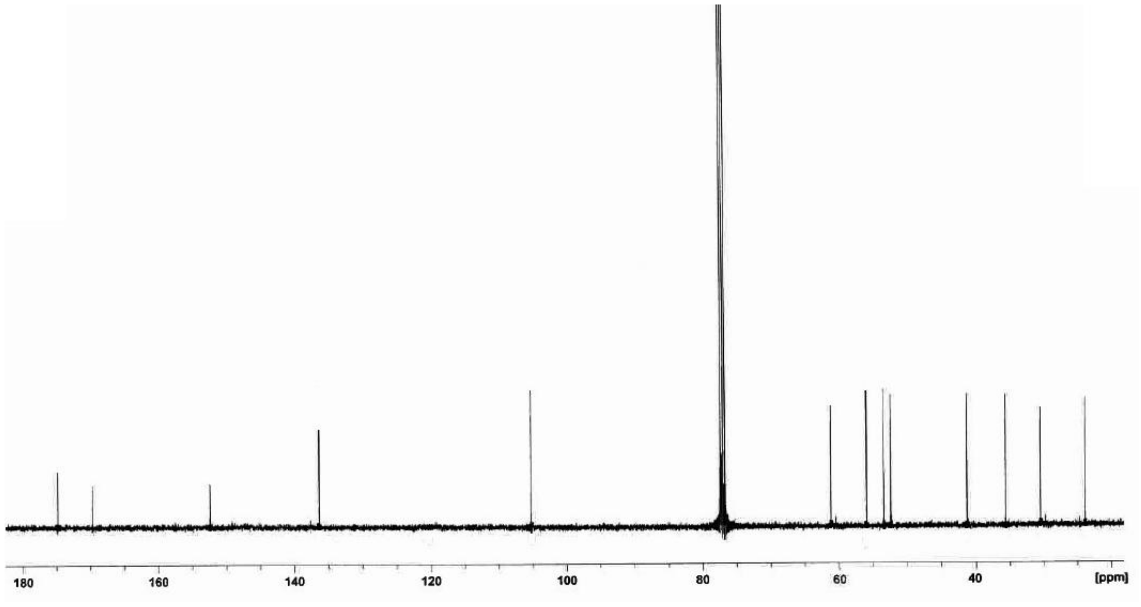

S6. ^{1}H and ^{13}C NMR spectra (400 MHz and 100 MHz, respectively, $CDCl_3$) of compound **5**.

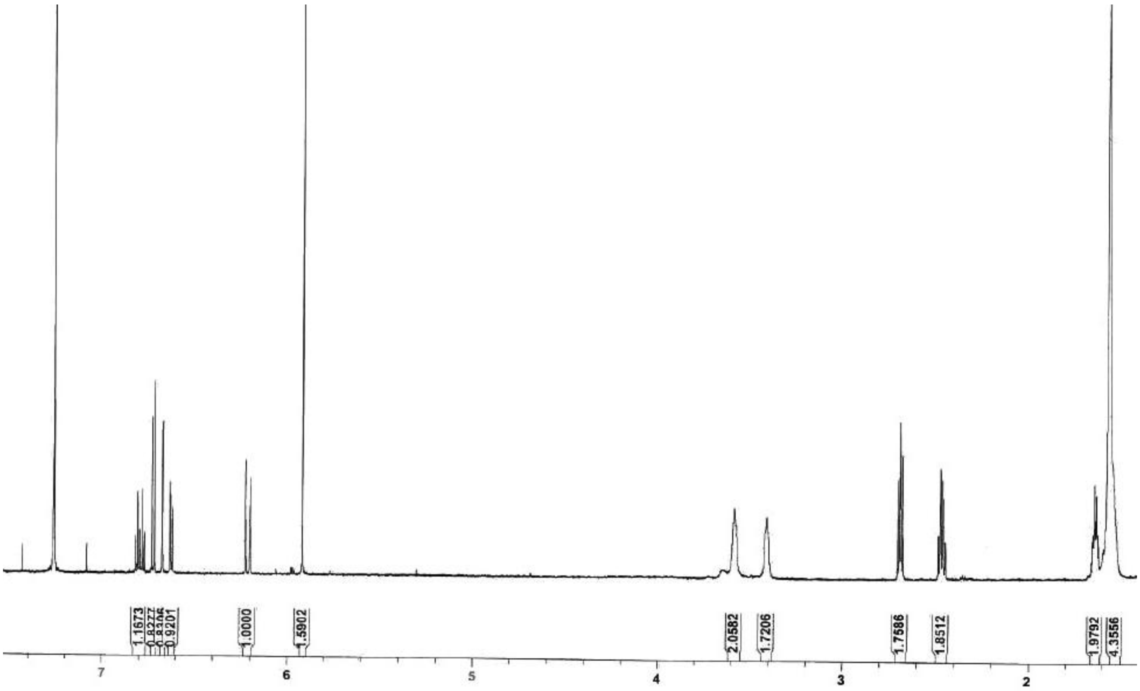

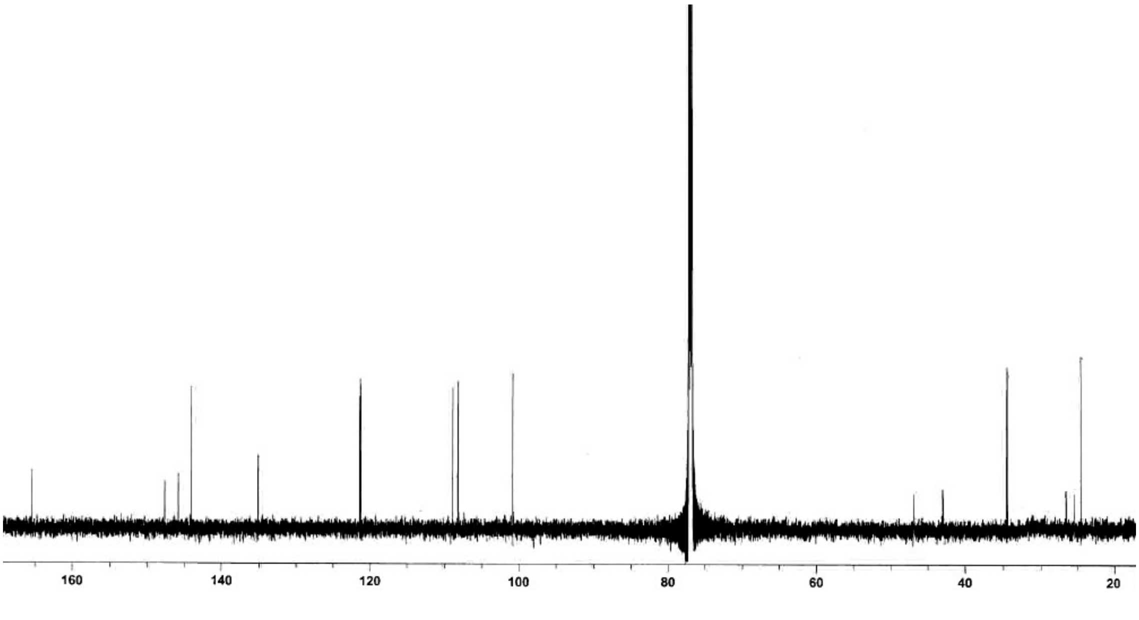

S7. ^{1}H and ^{13}C NMR spectra (400 MHz and 100 MHz, respectively, $CDCl_3$) of compound **6**.
